# Examining the Cross-Education Phenomenon in Lower Limbs: Insights from the Force–Velocity Profile

**DOI:** 10.3390/sports14020052

**Published:** 2026-02-03

**Authors:** Jessica Rial-Vázquez, Juan Fariñas, María Rúa-Alonso, Iván Nine, Manuel Avelino Giráldez-García, Eliseo Iglesias-Soler

**Affiliations:** Performance and Health Group, Faculty of Sports Sciences and Physical Education, Department of Physical Education and Sports, University of A Coruna, 15179 A Coruña, Spain; jessica.rial@udc.es (J.R.-V.); juan.farinas@udc.es (J.F.); maria.rua@udc.es (M.R.-A.); i.nine@udc.es (I.N.); manuel.avelino.giraldez.garcia@udc.es (M.A.G.-G.)

**Keywords:** cross-transfer, set configuration, unilateral training

## Abstract

This study explored whether the cross-education (CE) phenomenon could be examined through the force–velocity (FV) profile obtained from unilateral leg extension. Nineteen participants completed 5 weeks of unilateral knee extension interventions differing in set configuration. A traditional training group (TT) carried out four sets of 8 repetitions with 3 min of rest between sets, whereas an inter-repetition training group (IRT) completed 32 repetitions with 17.4 s of rest between repetitions. Exercise was performed with the 10-repetition maximum load on the dominant limb. Individual linear FV profiles (slope: S_FV_; theoretical maximum force and velocity: F_0_ and V_0_; and maximum estimated power: P_max_) were obtained for trained and untrained legs pre–post intervention. The trained limb showed significant increases in the post-test for F_0_, P_max_, and a steeper S_FV_ (*p* < 0.05). In the untrained limb, F_0_ (*p* = 0.042) and P_max_ (*p* = 0.010) also improved, whereas no changes were observed in V_0_ or S_FV_. Set configuration did not modulate the FV adaptations in the trained or untrained limb. CE was only observed for specific estimated force and power parameters. These findings indicate that strength and power transfer can be accomplished with low-fatigue training protocols, which may offer a more tolerable and practical option in clinical and performance settings.

## 1. Introduction

Cross education (CE) consists of the strength transfer from the trained to the untrained limb after a unilateral strength training period [1]. Neural adaptations have been suggested as the most plausible candidates to mediate the CE phenomenon [2,3,4] because it occurs without muscle hypertrophy or significant muscle activity in the non-trained limb [5]. It occurs with voluntary, stimulated, or imaginary contractions, and the magnitude of transfer has been estimated at around 12% [6]. This phenomenon has been shown to be useful for restoring symmetry in hemiparesis after strokes and during immobilizations caused by limb injuries [7].

Recently, it has been suggested that 2–3 weeks of unilateral resistance training are enough to cause CE [8,9], but expert consensus has recommended at least 4 weeks to enable a functionally significant transfer of strength [10]. Current research shows that the combination of fatigue with high-intensity loads produces greater cross-education than lower intensities [11,12,13]. For example, when comparing an elbow flexion unilateral protocol of 30 sets of 1 repetition at a 10-repetition maximum (10RM) load with another consisting of 5 sets of 6 repetitions at the same 10RM load (both matched for total volume and rest intervals), only the latter protocol, involving repetitions performed near muscular failure, produced cross-education [11]. In this line, Colomer-Póveda et al. [13] observed that training to failure with 25% of 1RM did not induce CE, whereas training with 75%, whether or not muscular failure was reached, resulted in CE in both cases.

CE is commonly explored through specific tests such as maximum voluntary isometric contraction, 1RM load, peak torque, pennation angle or muscle thickness [5] because they can identify significant changes in strength manifestation. However, to the best of our knowledge, no studies have explored more comprehensive strength evaluations. Establishing the force–velocity relationship (FV) allows for the determination of different indicators of muscle function and provides a more integrative mechanical representation of an individual’s capabilities. In this sense, recording individual FV profiles may provide information summarized by theoretical maximal force (F_0_), velocity (V_0_), the maximal power output (P_max_) and the slope of the linear regression (S_FV_). The literature suggests that individual FV profiles are sensitive to change after a few weeks of resistance training, and training protocols have the potential to impact different regions of that relationship [14]. In this sense, the manipulation of the resistance training parameters is key to producing changes in a specific part of the FV spectrum. Particularly, the set configuration parameter, defined as the number of repetitions performed in each set with regard to the maximum feasible, has been suggested to modulate the FV adaptations after middle-term interventions [15]. Training programs incorporating short set configurations, commonly referred to as cluster sets [16], performed far from muscular failure, have demonstrated particular efficacy in shifting the FV relationship toward a velocity-oriented profile in lower limbs (i.e., flatter slopes) [15]. Cluster set protocols have been associated with minimal intra-set velocity loss, no significant elevations in blood lactate concentration, and a low rate of perceived exertion, factors that collectively enable individuals to sustain exercise performance without experiencing substantial levels of fatigue [17,18]. Given that CE is primarily driven by neural adaptations, it can be hypothesized that more fatiguing long set configurations may be especially effective in eliciting adaptations in the nontrained limb, compared to shorter, cluster set configurations. However, to the best of our knowledge, no study has examined the entire FV spectrum to determine whether different set configurations elicit specific adaptations in the high-velocity or high-force region of the untrained limb.

Knowing that the manifestation of CE varies according to the manipulation of set configuration [11], this study aimed to determine the extent to which CE is reflected in the FV profile. In this context, the main aim of this study was to explore the FV profile changes in both trained and untrained limbs following unilateral knee extension programs differing in set configuration.

## 2. Materials and Methods

### 2.1. Experimental Design

**Figure 1:**
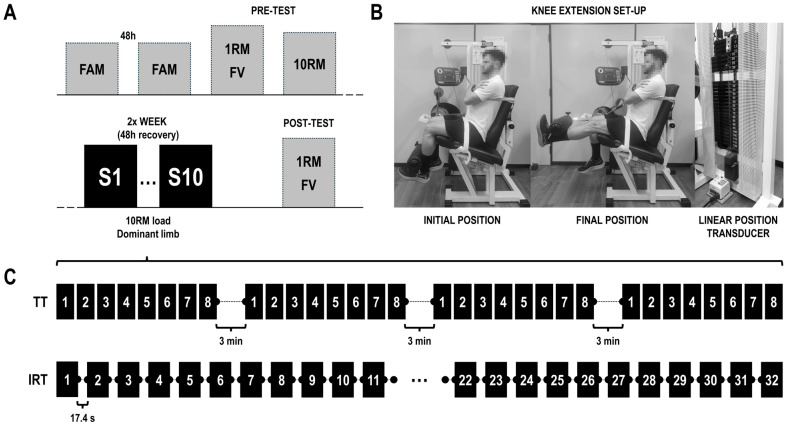
(**A**) Study design; (**B**) knee extension execution and set-up; (**C**) resistance training protocols. FAM: familiarization session; 1RM-FV: dynamic progressive loading test until one repetition maximum to obtain the force–velocity profile of each subject; 10RM: ten repetition maximum test; S1: resistance training session number one; S10: resistance training session number ten; TT: traditional training group; IRT: inter-repetition rest training group.

A randomized trial was conducted to address the purposes of the study. Figure 1 shows the experimental design and the resistance training protocols conducted during the intervention. Two preliminary sessions were provided to familiarize participants with the testing procedures. A dynamic progressive loading protocol was then executed for unilateral knee extension of the dominant and non-dominant leg, progressing until 1RM was determined. Moreover, a 10RM test was also conducted only with the dominant limb. Participants were then randomly assigned to two groups: a traditional training group (TT; *n* = 11) or an inter-repetition rest training group (IRT; *n* = 8).

The experimental groups completed ten sessions of unilateral knee extension exercises using the dominant limb at the 10RM load. Training was performed twice per week, with a 48 h recovery period between sessions. After the training intervention, the dynamic progressive loading test was repeated with the trained and untrained legs.

### 2.2. Participants

Nineteen healthy young participants (4 women and 15 men) completed all study procedures. They were recruited from the Faculty of Sport Sciences through informational forms distributed in academic courses and posters displayed throughout the faculty facilities. Inclusion criteria were the following: at least 3 months of regular resistance training experience (≥2 sessions per week); no contraindications for high-intensity exercise and no musculoskeletal injuries in the lower limbs or spine within the previous 12 months. Individuals who did not meet these criteria or who reported any condition that could interfere with training or testing were excluded. During all the procedures, regular communication helped maintain participant monitoring and study adherence. Moreover, risk-management procedures were applied throughout all testing and training sessions, including a standardized warm-up, professional supervision, and instruction in proper exercise technique. All participants were covered by the University of A Coruña’s student insurance, which provides medical assistance in case of injury during academic or research activities.

Descriptive data of the participants regarding their group allocation are shown in Table 1. An informed consent was read and signed by participants prior to enrollment. The study was approved by the University of A Coruna (Spain) Ethics Committee and conducted in accordance with the Declaration of Helsinki. Confidentiality was maintained by assigning coded identifiers to all data, which were stored securely on password-protected institutional servers with access restricted to the research team.

### 2.3. Procedures

#### 2.3.1. Familiarization Sessions

Two familiarization sessions with at least 48 h of rest between them were conducted in order to standardize knee extension machine positions for each participant, familiarize them with the exercise technique and determine their dominant leg. At the beginning of the first session, body mass and height were assessed using a digital scale (Omron BF-508, Omron Healthcare Co., Kyoto, Japan) and a stadiometer (Seca 202, Seca Ltd., Hamburg, Germany). Body mass index was calculated as kilograms divided by height in meters squared (kg/m^2^).

The sessions then continued with a standardized warm-up of 5 min of cycling on a cycle ergometer (Monark 828E, Monark Exercise AB, Vansbro, Sweden) at 60–80 rpm. During each session participants were asked to complete one set with their perceived 50% maximum load and two sets to approximate the 10RM load. The final set was completed until muscular failure to familiarize participants with maximal effort. Rest time between sets was 2 min. Familiarization was conducted with both limbs.

#### 2.3.2. Knee Extension Exercise

The exercise was performed unilaterally on a knee extension machine (Technogym, Gambettola, Italy) with 90° on hip and knee flexion. The lever arm and backrest were fixed in the same position for all testing and intervention sessions. Participants were secured with straps by the hip and chest in order to isolate the knee extensor muscles. Arms were folded across the chest. The range of motion was from 80° (knee flexed) to 0° (full extension).

#### 2.3.3. 1RM Test

A 1RM load was measured using a progressive load protocol based on velocity loss. Initially, participants completed 5 min of cycling at 60–80 rpm followed by 5 min of joint mobility exercises. They then performed 10 controlled repetitions of unilateral leg extension using a self-selected light load. The test then began with participants performing three repetitions at 20 kg as fast as possible. The greatest mean propulsive velocity (MPV) of those three repetitions was set as the maximum reference velocity. Sets of three repetitions were repeated (with 1 min of rest between them) until participants lost 25% of the maximum reference velocity. Load increments ranged from 10 to 5 kg. After this, participants performed two repetitions per set with increments of 5–10 kg until losing 50% of the reference velocity. They then completed sets of one repetition with a 3 min rest using increments of 2.5–5 kg until 1RM was obtained, defined as the load at which participants were able to perform only one repetition. A linear velocity transducer (T-Force System, Ergotech Consulting, Murcia, Spain) was used in all sessions to obtain MPV, mean propulsive force (MPF), and mean propulsive power (MPP). The device was placed next to the weight stack of the leg extension machine, with its cable attached to a small metal protrusion on the top plate, ensuring that the sensor accurately tracked the vertical displacement of the load during each repetition. This procedure was performed on both the dominant and non-dominant legs, with the testing order randomized at pre-test and replicated at post-test. The procedure was performed before and after the 10-week protocol. For each participant, the FV relationship was calculated for both legs using MPV and MPF values recorded during this progressive loading test.

#### 2.3.4. 10RM Test

Participants were tested with the maximal load they could complete for 10 repetitions to failure, but not 11. Following a warm-up consisting of 5 min of cycling at 60–80 rpm and 5 min of joint mobility exercises (hip rotations, arm swings, ankle circles, and dynamic knee flexion–extension), participants completed a set with a load corresponding to 50% of 1RM as a specific warm-up. After 5–6 min of rest, they repeated the exercise with 70% 1RM. If the participants completed 11 repetitions, the load was increased by 2.5–5 kg, whereas if they could not complete 10 repetitions, the load was decreased until the 10RM was obtained. Muscular failure was identified when the participant was unable to overcome the load or when the full range of movement of the exercise was not completed. All the tests were recorded in 3 ± 1 attempts. This procedure was performed with the dominant leg, and the obtained load was used in the training sessions.

#### 2.3.5. Training Protocols

Participants were assigned to the TT or IRT groups following a randomized block design. Participants completed 10 unilateral resistance training sessions targeting the knee extensors of the dominant limb twice per week, with at least 48 h of rest between each session. The TT group carried out four sets of 8 repetitions with 10RM load and three min of rest between each set, whereas the IRT group completed 32 repetitions with the 10RM load and 17.4 s of rest between them (See Figure 1).

### 2.4. Data Analysis

From each progressive loading 1RM test (pre- and post-intervention for the dominant and non-dominant legs), the individual FV relationship was obtained considering MPV and MPF. In cases where multiple repetitions were recorded for a given load, the repetition exhibiting the greatest MPV was retained for analysis [19]. To establish the FV relationship, we used nine to fourteen data points, each representing the fastest repetition completed at a given external load. Linear models are commonly used to fit the FV relationship of resistance exercises due to their good reliability and goodness of fit [20]. Moreover, previous studies involving unilateral knee extension exercises have employed and recommended the use of linear models to fit FV data [21]. Accordingly, our data was analyzed using linear approaches.

Firstly, the goodness of fit of the linear model used to adjust FV data was presented through the coefficient of determination (R^2^) and the standard error of estimation (SEE). After obtaining the individual linear regression, the following parameters were obtained: the theoretical maximum force achieved at zero velocity (F_0_), the theoretical maximum velocity when force is zero (V_0_), the slope of the linear regression (S_FV_ = −(V_0_/F_0_)) and the theoretical maximum power (P_max_ = (F_0_ × V_0_)/4).

Post hoc power and sensitivity analyses were conducted using a statistical power analysis program (G*Power software: version 3.1.9.6, Dusseldorf, Germany). Statistical power for the within–between interaction in a repeated-measures ANOVA with two groups and two measurement points, assuming a total sample size of 19 participants, a correlation of 0.7 between repeated measures, and a medium effect size (f = 0.25), was estimated at 0.76. In addition, a sensitivity analysis was performed to determine the minimum detectable effect size for this interaction at an alpha level of 0.05 and a target power of 0.80. Under these assumptions, the analysis indicated that the design was sensitive to detect a medium effect size (f = 0.26).

### 2.5. Statistical Analysis

Descriptive data are reported as mean ± SD. For R^2^, medians and range are presented. Prior to inferential analysis, the assumption of normality was assessed for the study variables. Normality was evaluated using the Shapiro–Wilk test, which is recommended for small sample sizes.

Changes in FV profile parameters (F_0_, V_0_, S_FV_, and P_max_) were analyzed by 2 × 2 ANOVA with group (TT and IRT) and time (pre-test and post-test) as factors regarding trained and untrained legs. When a significant interaction was detected, post hoc tests were carried out with Bonferroni’s adjustment. The effect size for each factor of ANOVA was reported using the partial eta squared (pη^2^).

Statistical analysis was performed with SPSS 20 (IBM, Armonk, NY, USA) and graphical results were presented using Graphpad Prism v5.01 for Windows (Graphpad Software, San Diego, CA, USA). The level of statistical significance was set at 0.05.

## 3. Results

**Figure 2:**
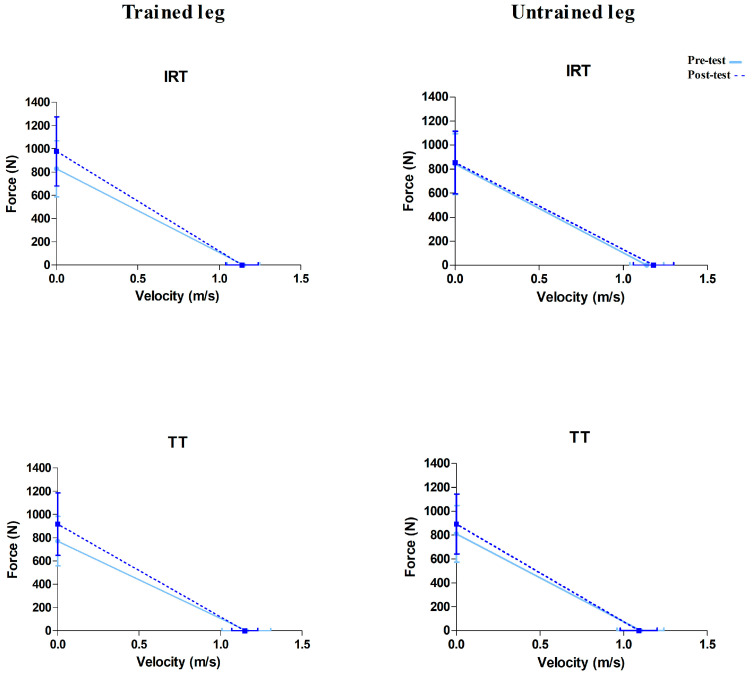
Mean force–velocity relationships of inter-repetition (IRT) and traditional (TT) groups before (solid line, light blue) and after training interventions (dashed line, dark blue). Data are displayed as means ± SD.

Regarding the pre-test, the medians of R^2^ for the trained and untrained legs were 0.972 (Range: 0.865 to 0.993) and 0.972 (Range: 0.926 to 0.992), respectively. The SEE values were 25.98 ± 11.84 N for the trained and 28.14 ± 13.39 N for the untrained leg. Considering the post-test, R^2^ for the trained and untrained legs were 0.966 (Range: 0.927 to 0.986) and 0.960 (Range: 0.919 to 0.991). The SEE values in the post-test were 36.44 ± 12.48 N for the trained and 30.65 ± 15.58 N for the untrained legs. Table 2 and Table 3 show pre–post values (mean ± standard deviations) and ANOVA results. Overall, significant increases in the post-test were observed in both the trained and untrained legs regarding F_0_ and P_max_, whereas only S_FV_ was improved after intervention in the trained leg (*p* = 0.001; pη^2^ = 0.495). Figure 2 represents the mean force–velocity relationships before and after intervention in both groups (i.e., TT and IRT) and both legs (i.e., trained and untrained).

## 4. Discussion

The FV relationship has relevant implications for muscle and exercise physiology, yet it remains unclear how different resistance-training set configurations influence the full FV spectrum, particularly in untrained limbs. Although previous studies have examined unilateral training effects, no research has specifically explored whether manipulating proximity to failure elicits distinct adaptations across the high-force and high-velocity regions of the FV profile. This gap is clinically relevant, since our findings can help to design resistance training protocols for rehabilitation following limb injury. Therefore, the aim of this study was to investigate FV profile changes in both trained and untrained limbs after unilateral knee extension programs differing in set configuration.

The main findings were: (i) significant improvements in F_0_, P_max_ and steeper slopes (S_FV_) observed in the trained limb, showing a change towards a force-oriented profile; (ii) increases in F_0_ and P_max_, but not in V_0_ or S_FV_, were detected in the untrained leg; (iii) set configuration did not modulate the changes in the FV parameters in either the trained or untrained legs.

The linear regression models exhibited an excellent goodness of fit (R^2^ > 0.870) when analyzing force and velocity data for both trained and untrained limbs. These findings reinforce the suitability of this regression approach for modeling FV relationships in unilateral exercises [20,21].

### 4.1. Trained Leg

After the intervention, the FV profile of the trained leg shifted toward a force-oriented profile (i.e., steeper slopes). The increase in F_0_, together with the absence of changes in V_0_, indicates that the enhancement in P_max_ was primarily driven by substantial adaptations in the high-force region. This change was consistent in both training groups, indicating that set configuration did not modulate the changes in the FV profile. This aligns with the findings of a previous study [22], in which two unilateral knee extension resistance training protocols were conducted separately on each leg (inter-repetition vs. traditional set configuration). Despite the greater training velocities observed during the inter-repetition rest protocol, both approaches led to similar improvements in the FV mechanical profile, specifically toward greater force-generating capabilities. On the other hand, our results contrast with other studies that showed different neuromuscular adaptations when resistance training protocols differing in set configuration were performed [15]. Short set configurations produced improvements in the high-velocity region of the FV profile, attributable to their capacity to preserve neuromuscular performance during training (i.e., minimal intra-set losses in velocity and power), even when performed with high load intensities [15]. Specifically, that study reported a shift toward a more velocity-oriented profile only after short set configurations in the lower-limb exercise (i.e., half squat), whereas this adaptation was absent in the upper-limb exercise (i.e., bench press).

In this sense, the velocity-specificity principle of resistance training indicates that strength gains are greatest at or near the velocity of training [23]. Based on this, previous studies have shown that adaptations in the high-velocity region of the FV relationship are primarily produced after explosive training with medium-light loads, whereas heavy-load training preferentially enhances the high-force region [24]. In our study, load intensity was equated between protocols (i.e., 10RM load). This load a priori should stimulate the medium- to high-force region of the FV profile, especially in the traditional training group, where the final repetitions of each set were performed near muscular failure. In this sense, it was not surprising that the TT group shifted towards a more force-oriented profile. A similar outcome was observed following the IRT protocol, where only minimal velocity losses are presumed to have occurred. The adaptations observed may be explained by the execution of the IRT intervention, which did not benefit from the stretch-shortening cycle. Each repetition required initiating the movement from rest and overcoming inertia to generate contraction (i.e., concentric pattern).

### 4.2. Untrained Leg

To the best of our knowledge, this is the first study to explore whether CE could be examined through the FV profile. Although the FV relationship can be described using different parameters depending on the regression model applied to the FV data, the slope remains the primary parameter accounting for individual FV profile shifts [25]. Our results showed significant improvements in F_0_ and P_max_ in the untrained leg, but no changes in S_FV_ were observed. In this regard, CE appeared to manifest only in specific estimated force- and power-related parameters, without producing a measurable modification in the slope. This suggests that, while certain mechanical capacities may transfer contralaterally, such transfer may not be sufficient to alter the overall FV profile structure.

The overall magnitude of CE is estimated to be around 12% of the baseline force value for concentric contractions [6]. The recommended exercise pattern to maximize the strength gains in the contralateral limb includes isometric (>80% of maximum voluntary isometric contraction), eccentric or eccentric–concentric actions (>80% 1RM) [26]. Overall, F_0_ in the trained leg increased by 148.18 N (pη^2^ = 0.628), corresponding to an 18.5% improvement. In the untrained leg, F_0_ increased by 47.3 N (pη^2^ = 0.221), representing a 5.7% change. However, this improvement was not sufficient to modify the FV slope in the untrained leg. That improvement in F_0_ also contributed to enhancing P_max_ in the untrained leg to a similar magnitude (i.e., 16.1% improvement in the trained leg vs. 6.4% increase in untrained leg). As previously noted, the exercise pattern in the trained limb was exclusively concentric in the IRT group, which may have limited the magnitude of strength transfer. It remains unclear whether an eccentric–concentric training pattern performed at higher load intensities and over a longer intervention period (i.e., more weeks, sessions, and total volume) [13,26] could elicit greater neural adaptations that ultimately enhance F_0_ to a degree sufficient to modify the slope of the FV relationship and, consequently, shift the individual profile.

The improvements observed in the high-force region reinforce the utility of maximum voluntary isometric contractions to explore the CE phenomenon. Moreover, the increments observed in P_max_ suggest that power-oriented tests may represent a valuable additional tool to investigate CE. Although muscular power has not traditionally been one of the most extensively investigated variables in the context of cross-education, some studies have nonetheless examined this parameter and provided empirical support for the power gains observed in the untrained limb in the present study. For example, a study assessing knee extensors and flexors during isokinetic testing at 240°/s and 60°/s reported that power adaptations were transferred from the trained to the untrained limb, with greater gains observed in the knee extensors than in the flexors [27]. Another study examined the effects of eccentric-overload isoinertial resistance training leg protocols on the adaptations in power measured at 40–80% 1RM and unilateral vertical jump height. Authors reported significant increases in the untrained limb for unilateral vertical jump height (6.0–32.9%) and muscle power (6.8–17.5%) [28]. The authors also suggested that lower percentages of eccentric overload were associated with greater increases in muscle power. An increase in muscle power in non-trained limbs may be particularly relevant in other population groups, such as older adults. Many fundamental daily activities (e.g., walking, rising from a chair) rely on the capacity to generate force rapidly, and therefore power adaptations have the potential to enhance mobility-related outcomes in the elderly. In this sense, CE has clear clinical relevance for frail populations, as fractures caused by falls or the sequelae of cerebrovascular accidents often result in unilateral immobilization or substantial functional deficits. Given that muscular power is a key determinant of training performance, CE may help maintain, or even enhance, this parameter when an injury or immobilization affects one limb. Such preservation of power in the affected side could potentially facilitate an earlier return to sport participation and daily functional activities compared with traditional rehabilitation protocols [26].

This study is not without limitations. First, the unbalanced sex distribution in the sample limited the examination of potential sex-related differences. Future studies with larger and more balanced samples of men and women would be valuable to further explore CE through the FV profile while considering possible sex-specific adaptations. A recent review suggested that women may benefit more from cross-education in terms of muscle strength; however, this observation requires further sex-specific comparisons to be confirmed [5]. Second, the intervention comprised only 10 training sessions, which may have constrained the magnitude of strength adaptations observed in the non-trained limb. Third, the IRT group performed exclusively concentric contractions, a factor that could have attenuated the strength transfer, given that eccentric training has been shown to elicit greater CE responses. Lastly, we did not include a comparison control group. It is important to note that the participants were physically active students engaged in various sports, making it difficult to control their external physical activity. This could have introduced unintended variability in a potential non-training control group, which ideally should maintain a true “no-exercise” condition.

From a practical standpoint, our results indicate that the slope of the FV profile did not change in the untrained limb following a 10-session unilateral resistance-training program performed at the 10RM load. Several plausible factors may explain the absence of an adaptation in the FV profile slope. Training variables such as load intensity, contraction pattern, and the relatively short duration of the intervention may not have been sufficiently demanding to elicit measurable shifts in this parameter. Nevertheless, increases in the theoretical maximal force (F_0_) and maximum estimated power (P_max_) were observed in the untrained limb, regardless of set configuration. These findings suggest that strength and power transfer can be achieved through training protocols performed far from muscular failure (i.e., short sets). Given that similar outcomes were obtained regardless of proximity to failure, implementing low-fatigue, low-perceived-exertion protocols may be advantageous for participants, as they could be better tolerated by populations such as older adults or individuals undergoing rehabilitation following limb injury.

## Figures and Tables

**Table 1:** Participants’ characteristics.

Parameter	Traditional Training Group	Inter-Repetition Training Group
Age (years)	23 ± 3	22 ± 2
Sex	2 females/9 males	2 females/6 males
Height (cm)	172 ± 7	174 ± 8
Body mass (kg)	73 ± 9	74 ± 11
BMI (kg·m^−2^)	25 ± 2	24 ± 2
Laterality	2 left-footed/9 right-footed	1 left-footed/7 right-footed

BMI: body mass index.

**Table 2:** Pre–post intervention values (mean ± standard deviations) and ANOVA results in both groups (i.e., TT and IRT) considering the untrained leg.

									ANOVA *p*-Value and (pη^2^)		
	Parameters	Group	Pre-Test			Post-Test			TIME	GROUP	T × G
UNTRAINED LEG	S_FV_ (N·s/m)	IRT	−735.12	±	69.30	−727.79	±	76.36	0.254 (0.076)	0.650 (0.012)	0.174 (0.076)
		TT	−735.48	±	59.10	−815.60	±	65.12			
	F_0_ (N)	IRT	841.47	±	253.41	855.82	±	260.52	0.042 (0.221)	0.979 (0.000)	0.129 (0.130)
		TT	810.20	±	226.79	889.26	±	250.98			
	V_0_ (m/s)	IRT	1.14	±	0.10	1.18	±	0.12	0.624 (0.014)	0.213 (0.090)	0.263 (0.073)
		TT	1.10	±	0.14	1.09	±	0.11			
	P_max_ (W)	IRT	242.06	±	81.44	253.49	±	82.26	0.010 (0.328)	0.741 (0.007)	0.512 (0.026)
		TT	226.28	±	78.23	244.64	±	77.81			

S_FV_: slope of the linear force–velocity regression (negative values reflect the inverse FV relationship; x-axis = velocity, y-axis = force); F_0_: force axis intercept; V_0_: velocity axis intercept; P_max_: maximum estimated power; IRT: inter-repetition group; TT: traditional training group; T × G = time × group interaction.

**Table 3:** Pre–post intervention values (mean ± standard deviations) and ANOVA results in both groups (i.e., TT and IRT) considering the trained leg.

									ANOVA *p*-Value and (pη^2^)		
	Parameters	Group	Pre-Test			Post-Test			TIME	GROUP	T × G
TRAINED LEG	S_FV_ (N·s/m)	IRT	−711.80	±	178.17	−853.96	±	256.93	0.001 (0.495)	0.545 (0.022)	0.939 (0.000)
		TT	−658.91	±	153.88	−795.78	±	230.99			
	F_0_ (N)	IRT	827.20	±	240.50	977.81	±	296.86	<0.001 (0.628)	0.614 (0.015)	0.931 (0.000)
		TT	770.59	±	212.12	916.34	±	269.52			
	V_0_ (m/s)	IRT	1.15	±	0.10	1.14	±	0.09	0.637 (0.013)	0.859 (0.002)	0.936 (0.000)
		TT	1.16	±	0.15	1.15	±	0.09			
	P_max_ (W)	IRT	241.73	±	82.65	281.03	±	88.06	<0.001 (0.597)	0.700 (0.009)	0.853 (0.002)
		TT	228.46	±	78.01	264.92	±	82.21			

S_FV_: slope of the linear force–velocity regression (negative values reflect the inverse FV relationship; x-axis = velocity, y-axis = force); F_0_: force axis intercept; V_0_: velocity axis intercept; P_max_: maximum estimated power; IRT: inter-repetition group; TT: traditional training group; T × G = time × group interaction.

## Data Availability

The original contributions presented in this study are included in the article. Further inquiries can be directed to the corresponding author.
